# Fresh Basil Infusion: Effect of Sous-Vide Heat Treatment on Their Volatile Composition Profile, Sensory Profile, and Color

**DOI:** 10.3390/molecules27010005

**Published:** 2021-12-21

**Authors:** Artur Głuchowski, Ewa Czarniecka-Skubina, Krzysztof Tambor, Elvyra Jariené

**Affiliations:** 1 Department of Food Gastronomy and Food Hygiene, Institute of Human Nutrition Sciences, Warsaw University of Life Sciences (WULS), 02-778 Warsaw, Poland; artur_gluchowski@sggw.edu.pl (A.G.); krzysztof_tambor@sggw.edu.pl (K.T.); 2 Institute of Agricultural and Food Sciences, Agriculture Academy, Vytautas Mangus University, LT-53361 Kaunas, Lithuania; elvyra.jariene@vdu.lt

**Keywords:** sous-vide, basil, infusion, sensory quality, volatile compounds profile, color

## Abstract

Herbs, including basil, are used to enhance the flavor of food products around the world. Its potential is influenced by the quality of fresh herbs and processing practices, wherein conditions of heat treatment play an important role. The aim of the research was to determine the effect of sous-vide heat treatment on the volatile compounds profile, sensory quality, and color of basil infusions. The material used for research was aqueous basil infusion prepared conventionally at 100 °C, and using the sous-vide method (65, 75, and 85 °C). The composition of volatile compounds was identified by GC/MS analysis, the sensory profile was assessed using a group of trained panelists, while the color was instrumentally assessed in the CIE Lab system. No significant differences were found in the intensity of the taste and aroma of basil infusions at different temperatures. Seventy headspace volatile compounds were identified in the analyzed samples, ten of which exceeded 2% of relative area percentage. The most abundant compounds were eucalyptol (27.1%), trans-ocimene (11.0%), β-linalool (9.2%), and β-myrcene (6.7%). Most of the identified compounds belonged to the terpenes and alcohols groups. Our findings show that the conventional herbal infusion was more like a sous-vide infusion prepared at the lowest temperature SV_65_, while SV_75_ and SV_85_ were similar to each other but different from the conventional. However, a smaller number of volatile compounds in the samples heated at higher temperatures of sous-vide were identified. The sous-vide samples showed a higher content of alkanes. The sous-vide method (*p* ≤ 0.05) resulted in darker, less green, and less yellow basil leaves than fresh and traditionally steeped ones. Long heat treatment under vacuum at higher temperatures causes a pronounced change in the aroma composition.

## 1. Introduction

Herbs play an important role as natural flavoring substances during food processing. Application of the herb maceration and the infusion processes, and a medium that is a solvent (e.g., water, alcohol, and fat), enable herb wines, herbal tea infusions, aromatized vinegar, and oils commonly used for products like bread or vegetables to be made [1,2,3,4,5,6]. Recently, the popularity of herb macerates (e.g., for flavored oils in cooking or herb syrups in mixology) in the foodservice industry, as well as herbal infusions among consumers, has significantly increased [7]. They are used not only for seasoning or hedonic reasons but also as a source of harmless natural antioxidants [8]. Herbal infusions are usually prepared by steeping the dried aromatic parts of plants such as roots, leaves, flowers, fruits, and other elements in hot or boiling water. Nowadays, many species of aromatic and medicinal plants such as chamomile, lemon balm, basil, peppermint, lemon thyme, lemongrass, or lemon verbena are used to produce these infusions [7,9]. Herbal infusions are widely used in the treatment of diverse disease conditions [10].

Basil (*Ocimum basilicum* L.) is an aromatic herb belonging to the *Lamiaceae* family that plays both a significant culinary and an ornamental role. It is an essential ingredient of Mediterranean and other cuisines due to its unique and pleasant aroma. In addition to its culinary application, basil is utilized by the pharmaceutical and cosmetic industries for its chemical composition [11,12,13], which mainly includes polyphenolic acids and flavonoids that exhibit antioxidant, antiviral, antibacterial, and therapeutic properties [13,14].

Food is a complex matrix that consists of protein, lipid, carbohydrate, and phenolic compounds as well as aroma compounds that interact with these components [15]. The basil aroma is a result of certain volatile compounds. These are classified into two main chemical groups: terpenoids and phenylpropanoids, which are produced in highly specialized structures known as peltate glandular trichomes (PGTs), located on the aerial parts of the plant, mainly the leaves [16,17]. The release level of the volatile compounds and other flavor constituents into the infusions decides their sensory quality [18]. The sensory quality of an infusion is affected by the quality of the herb used (soil and weather conditions, harvesting, processing, and storage practices), and the preparation practices (quality of water, mutual proportion of herb and solvent, steeping period, and temperature) [7,9].

Sous-vide is a method of cooking food in thermostable vacuumed pouches under strictly controlled temperature and time parameters, offering improved flavor, texture, and nutritional values along with extended shelf life in comparison with conventional cooking methods [19]. Only a few researchers [20,21,22] have studied the effect of the sous-vide method on the quality of herbs. Alcusón et al. [21] stated that Borage stalks processed with the sous-vide method were lighter and greener than conventionally cooked stalks and had increased phenolic content and antioxidant activity.

Although the use of herbs and spices in dishes is intended to add new sensory notes, the lion’s share of studies evaluated the bactericidal effect and the possibility to extend the shelf life of their oils on food of animal origin. The antimicrobial effect of rosemary and thyme oils [20] and sage oil [23] on *L. monocytogenes* in sous-vide beef as well as oregano oil [22] in sous-vide salmon was determined. Some studies investigated the effect of the herbs’ addition on the sensory and nutritional value of plant-origin products. The positive effect of rosemary oil on the sensory quality of sliced potatoes [24], as well as on vitamin C and polyphenols in potatoes [25], was stated. Only a few studies aimed to evaluate the effect of sous-vide on fresh herbal plants. Recently published works were dedicated to evaluating the sensory profile of meat products with basil extract [26], as well as an aromatic profile of pesto [12], or in essential oils from various basil species [27].

Despite this interest, no one, to the best of our knowledge, has evaluated the sensory and volatile profile of fresh basil on a neutral matrix and basil heat treated at various temperatures. Therefore, the aim of the study was to determine the effect of sous-vide heat treatment on the sensory profile, volatile compounds’ composition, and color of basil infusions in relation to the traditional method. A research hypothesis was made that the volatile compounds’ profile of basil aqueous infusions heated in hermetic pouches under strictly controlled parameters is richer than under traditional infusion methods.

## 2. Results

### 2.1. Volatile Compounds’ Profile of Basil Infusion Produced with Various Methods

The identified headspace volatile compounds and their relative area percentage are presented in Table 1. Seventy various volatile compounds were identified in the analyzed samples, ten of which exceeded 2% of relative area percentage (Table 1). Most of the identified compounds belonged to the terpenes and alcohols groups.

The highest number of volatile substances was identified in the samples of fresh herbs (97.7% of the area), and slightly less in the SV_65_ and T_100_ samples (93.5% and 93.8%, respectively). A smaller share was identified in the samples heated at higher temperatures; SV_75_ and SV_85_ (82.6–83.0%), which results from the significant number of high-molecular compounds from the package accumulated in the headspace. The most abundant compounds in basil infusions and fresh basil samples were eucalyptol (27.1%—the average of all samples), trans-ocimene (11.0%), β-linalool (9.2%), and β-myrcene (6.7%).

The profile of volatile compounds in the analyzed infusions differed significantly depending on the parameters applied (Table 1). Sous-vide infusions, unlike fresh basil samples, did not contain compounds responsible for the notes of freshly cut grass or leaves. The most notable were 3-hexene-1-ol, 1-hexanol, and 2-hexene-1-ol. However, they were detected in small amounts in traditional infusions. The exception was 3-hexene-1-ol, trace amounts of which (0.05%) were found in the SV_75_ sample. Moreover, fewer volatile compounds were identified in sous-vide aqueous infusions than in traditional infusions. Sous-vide samples treated at higher temperatures (SV_75_ and SV_85_) had a higher proportion of α-thujene, α-pinene, camphene, β-pinene, and α-terpinene (having piney and woody evocations) than the SV_65_ or traditional infusion samples. Basil infusions had a greater share of γ-terpinene, β-linalool, bornyl acetate, and eugenol (mainly sweet, floral, and spicy notes) than fresh basil. On the other hand, heat treatment negatively influenced the share of volatile compounds responsible for forest and wood notes in the profile. The most noticeable was the decrease in the content of β-farnesene and germacrene D. 

Traditional infusions, in contrast to those prepared using the sous-vide method, did not significantly reduce the share of compounds such as limonene, terpinolene with citrus notes, and estragole with licorice and anise notes. The content of copaene and β-element in the traditional infusion, although different from the content in the fresh basil, was statistically significantly higher than in the sous-vide infusions. The high initial temperature of the water and atmospheric conditions in the traditional infusion resulted in a significantly lower share of volatile compounds like eucalyptus and camphor. However, a greater share of β-myrcene, α-phellandrene, and trans-ocimene was found in them. In the sous-vide samples, in contrast to the others, alkanes (decane, dodecane, and tridecane) and octane, responsible for green, lemon, and soap notes, were identified. With the increasing temperature, an increase in the share of δ 3-caren and cis-ocimene was also determined.

### 2.2. Sensory Profile of Sous-Vide Basil Infusions

There was no significant effect of temperature on the odor and flavor profile assessed by panelists (Table 2). However, non-significant (*p* > 0.05) observation of a slight decrease in fresh and natural basil odor and flavor intensities were noted as the process temperature increase.

The color of basil infusions was also affected by temperature (*p* < 0.05). As the temperature of sous-vide increased, color intensity decreased. The color of traditional infusions was more intense than sous-vide infusions processed at 85 °C. 

The individual sensory characteristics of basil infusions are highly correlated with the selected volatile compounds as follows:

- Natural basil odor and herbal odor—1-octanol, respectively, r = 0.97 and r = 0.99;
- Sweet odor –β-Linalool (0.98), Borneol (0.97), α-Terpineol (0.96);
- Pungent odor—α-Terpinene (0.96), γ-Terpinene (0.99);
- Natural basil flavor—Dimethyl sulfide (0.99), Eucalyptol (0.97), Dodecane (0.96), Tridecane (0.98);
- Fresh flavor—Nonanal (0.98), α-Terpineol (0.95), β-Selinene (0.99);
- Herbal flavor—3-Octanone (0.99), Sabinene hydrate trans (0.96);
- Sweet taste—α-Terpineol (0.97);
- Bitter taste—3-Octanone (0.96);
- Astringent sensation—aftertaste—Dimethyl sulfide (0.98), Tridecane (0.96).

**Figure 1:**
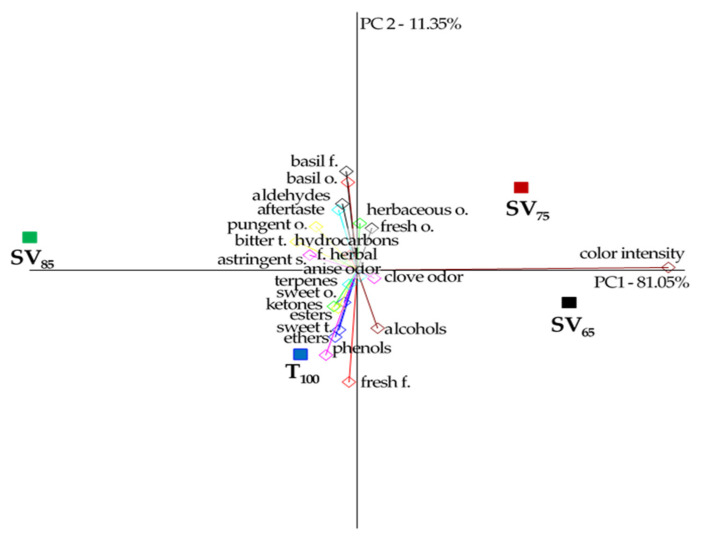
Principal components analysis (PCA) biplot of basil infusions (SV_65_, SV_75_, SV_85_, and T_100_).

The results of Principal Component Analysis (PCA) of the sensory and volatile profiles of basil infusions are presented in Figure 1. The first two principal components of PCA explained 92.4% of the total variability between the samples of basil infusions, but 81.05% of the variance was attributed to PC1, which was strongly associated with color intensity.

The second principal component demonstrates a relationship between flavor and odor intensity and the content of volatile compounds groups. The intensity of natural basil odor and flavor as well as aftertaste intensity was related to the aldehydes content and was negatively related to traditional basil infusions (T_100_), which is proven by the location of those attributes on the opposite side of the plot origin. Fresh flavor and sweetness were related the most with phenols and ethers content. These vectors were closely related to sample T_100_.

Generally, in terms of the volatile composition as well as flavor and odor intensities, a traditionally infused sample was more like a sous-vide infusion cooked at the lowest temperature; SV_65_, while SV_75_ was more similar to SV_85,_ as shown by the contrary location of these samples relative to the OX *X*-axis. It can be concluded that prolonged heat treatment in a vacuum at higher temperatures causes a pronounced change in aroma composition.

### 2.3. The Color of Infusions and Basil Leaves Measured Instrumentally

The instrumental color measurement of basil infusions performed immediately after their preparation revealed that along with the increasing temperature of the sous-vide method, the color of infusions became brighter (L*), and less red (a*) and yellow (b*). The color of traditional infusions was like SV_75_ (L*, a*) but significantly less yellow. Only the color of SV_85_ samples, did not change significantly after 2 h of storage under atmospheric conditions. In other cases, the color became darker, redder, and usually yellower (Table 3).

The color of basil leaves was affected by the heat treatment (*p* ≤ 0.05) and became darker, less green, and less yellow. With the temperature increase in the sous-vide treated samples, there was a proportional decrease in greenness, but not in lightness and yellowness. The color of conventionally steeped basil leaves was the least changed. Conventional samples were significantly darker but did not differ in yellowness and greenness from fresh leaves. The total color difference (ΔE) of sous-vide treated leaves (38.6–42.9) increased with the temperature and was higher than the traditionally infused (20.4). This is caused by a lower color chroma (C) of sous-vide samples (17.5–13.9) when compared to T_100_ samples (34.0). The most changed color from conventionally heated leaves had sample processed at the highest temperature of sous-vide SV_85_ (Table 3).

## 3. Discussion

### 3.1. Effect of Various Heat Treatment Methods on the Aroma Profile of Basil Infusions

Basil does not have one “right” aroma, and its aroma profile is heterogeneous [17]. Fresh basil aroma is a complex mixture of many volatile organic compounds (VOc) that are present in various proportions and have various odor activity values. Aroma quality depends on essential oil content and composition (mainly terpenoids and phenylpropanoids). The most abundant compounds in the aromatic profile of basil differed between authors: (Z)-3-hexenal, eucalyptol, linalool, and eugenol were indicated by Blank [29]; linalool, eucalyptol, and trans-bergamotene were pointed out by Ciriello et al. [12]; eucalyptol, linalool, and eugenol were reported by Ciriello et al. [11]; while linalool, eugenol, and cis-alpha-bergamotene, by Tirillini and Maggi [30]. In this study, the similar VOc of fresh basil (eucalyptol, linalool trans-ocimene, β-linalool, and β-myrcene) were dominant. These discrepancies in the most plentiful volatiles are normal, as four major essential oil chemotypes in *O. basilicum* were identified: estragole-rich; linalool-rich; methyl eugenol-rich; and methyl cinnamate rich [31]. The results of Sonmezdag et al. [4] suggest that the most aroma-active compounds in Iranian and Turkish basil were linalool and estragole, which were present in significant quantities in our study. The compounds present in smaller amounts also results in a decrease in similarity to fresh basil.

After heat treatment and independently of processes conditions, the volatile compounds profile was changed. In our study, the relative percentage content of eucalyptol in the sous-vide samples was higher than in both the fresh samples and the conventionally infused. The dynamics of the changes are entirely different than in drying, where high temperature caused evaporation of eucalyptol and many other VOc [32]. Unlike in this study, other authors [33] reported that the relative percentages of linalool and eucalyptol increased with increasing heating time and temperature, but they used oils with dried basil. 

The most abundant compound in this study was eucalyptol, of which the highest share was noted in SV_65_ and the lowest in traditional infusions. In the study by Rocha et al. [7] on aromatic compounds of lemon verbena, concentrations of eucalyptol and limonene decreased with temperature at the longer steeping times. In this study, heat treatment lowered the share of volatiles giving forest and wood notes in the profile, which is in accordance with the results of Wang et al. [34], who found that high temperature can decrease the floral, woody, fatty, and sweet notes, and enhance the odor of green, roast, and fruity scents. In this study, basil infusions had a greater share of volatiles responsible for sweet, floral, and spicy notes, e.g., β-linalool and eugenol. Although no dramatic changes caused by cooking the basil leaves were reported by Pojjanapimol et al. [35], they reported slightly elevated levels of eugenol, similarly as in that study.

Table 4 shows volatile compounds identified in basil infusions along with references confirming the high probability of their identification in basil. The compilation considers the results of research by various authors [33,36,37,38,39], in which the chromatographic analysis was carried out using a capillary consisting of a non-polar phase with 5% participation of phenyl groups. The largest number of confirmations in the literature concerns compounds occurring in herbs in significant amounts. Due to the fact that most of the chromatographic analyses were carried out on herbal extracts or extracts, the works of other authors did not identify aliphatic alcohols and aldehydes typical for freshly cut herbs (e.g., 3-hexen-1-ol, (E) -2 -hexenal). In the sous-vide samples, there was a higher relative content of alkanes.

Applied heat treatment, especially vacuum cooking, resulted in the degradation of volatile compounds associated with freshly cut grass or leaf notes. This is consistent with the results of Łyczko et al. [40], who found that even slight drying of fresh cilantro at 50 °C significantly decreases (*Z*)-Hex-3-en-1-ol and (*E*)-Hex-2-en-1-ol, and the effect is more notable with higher thermal treatment. The disappearance of almost all the compounds of the “green” note was not only observed during the drying process but also for blanching of the basil [41].

### 3.2. Effect of Various Heat Treatment Methods on the Sensory Profile of Basil Infusions

Herbs play an important role as a flavoring agent in food technology. The assessment of the influence of the sous-vide method on the sensory profile of herbs was not easy and required the use of a neutral food matrix (water), which would absorb flavor compounds. The use of foods as carriers, due to their complexity (protein, fats, carbohydrates, water, etc.) and interaction between food ingredients, could make the interpretation of the results even more difficult. For these reasons, it was decided to evaluate the influence of temperature on the composition of volatile compounds and to relate the results to flavor profiles using an aqueous matrix. 

The phase (water or steam state) in which aroma compounds are located contributes to a better perception of odor. Heating a substance in the mouth changes its state from water to steam and improves the perception of smell [42]. The flavor sensations emerge as a complex process, the first stimulus substances are already released in the mouth, then by retronasal olfaction, aromas are transported from the oral cavity to the nasal cavity, where they connect with the olfactory receptors. During the consumption of products, many physicochemical changes take place in the oral cavity, and volatile compounds are also released. This process is influenced by the components of saliva, e.g., proteins, teeth, and tongue [43,44].

This study did not demonstrate a significant effect of sous-vide cooking temperature on the odor and flavor profile evaluated by panelists. The reason for this is a long infusion time that leads to blurring the differences in intensities or not sufficiently differing parameters of the process. Although significant differences were detected using chromatographic methods, trained and experienced experts could not perceive them.

No significant differences in hedonic assessment of kenaf leave tea with different temperatures and infusion times were reported by Chong and Nyam [45]. On the other hand, a significant effect of temperature on the odor and flavor of herbs was shown in the research by Abo et al. [46]. These authors showed that oils macerated with basil or oregano at 60 °C were characterized by a higher liked odor (in the case of oregano, also flavor) than those prepared at 20 °C. However, the opposite tendency was observed in the case of the desirable taste of rosemary oil. Moreover, it was found that herbal oils with a more intense aroma (rosemary) were rated higher in terms of overall appearance, color, taste, and smell. 

More pronounced changes in flavor and odor intensity are visible during the drying process. Basil leaves that were oven-dried at a mild temperature of 60 °C reached the highest score for aroma, due to the least degradation of the aroma-flavor compounds [47]; however, the authors carried out the process without the use of water, and the research conditions were different than in this study.

Higher temperatures of other types of basil heat treatments, such as drying, affect the sensory quality of basil. It usually elicits a decrease in fresh, floral, and herbaceous odor intensity, while increasing spicy, hay-like, sweet, earthy, and woody intensity [48]. The applied temperature influences the efficiency of extraction by modulating the physicochemical properties of water. Higher temperature tends to lower the polarity of the water, increasing the solubility of the less polar compounds in the water [49].

In our research, although not significant, the intensity of the astringent sensation was slightly more notable in basil infusions prepared at a higher temperature, while it was fresh odor and flavor as well as basil flavor in infusions seeped at a lower temperature. This is in accordance with the findings of Lee and Chambers [50], who found that bitterness and astringency become stronger while green-related attributes become weaker as the brewing time and water temperature of green tea increases. This is also confirmed by the results of Miller et al. [51], who found that heat treatment of 2 h at 80 and 90 °C reduced the green/grass flavors’ intensity to negligible levels. 

Bitterness may be related to the presence of flavonoids and isoflavonoids in food. Dent et al. [52], who investigated the effect of solvents, temperature, and time on the content of phenolic compounds in sage, found that water extraction at 90 °C was associated with a higher content of polyphenolic compounds than extraction at 60 °C. Moreover, the content of the aforementioned compounds increased with the increase in the processing time (from 30 to 90 min). Therefore, it seems that the time of the treatment is as important as the temperature. The extended cooking time in the water medium had a positive effect on the content of flavanone glycosides and phenolic acids. 

In this research, bitter taste intensity slightly but insignificantly increased with increasing heating temperature; the heating time was 1 h, which also imitates the sous-vide heating process. Rocha et al. [7] obtained different results. The optimal combination of steeping for 6 min at a temperature of 96 °C, when preparing lemon verbena infusions, helps maximize of overall liking, high yield, high content of some aromatic, antioxidant, and phenolic compounds, while maintaining at a low level the extraction of the more astringent and bitter compounds, such as chlorogenic acid and epigallocatechin gallate [7].

There are no original studies in the literature on the influence of the maceration temperature of fresh herbs on the profile of volatile compounds. The authors indicate a significant influence of phenolic compounds on the aroma of herbal tinctures and beer [53,54], and herbal infusions (chamomile, St. John’s wort) depending on temperature (cold, or boiling water) and brewing time [55]. 

### 3.3. Effect of Various Heat Treatment Methods on the Color of Basil Leaf Infusions

The color of the basil varies depending on the part it comes from: leaf, stem, flower, or spike [56]. This study used only basil leaves.

The color of basil infusions measured shortly after their preparation became darker, redder, and yellower along with increasing steeping time. This is in accordance with the results of Marete et al. [57], who found that samples of feverfew (*Tanacetum parthenium*) aqueous infusions prepared at a temperature of 20–70 °C were darker and had a higher hue angle than those prepared at higher temperatures (80–100 °C). As they concluded, this was not related to the content of bioactive compounds because significant increases in the total phenols content were seen at temperatures above 75 °C. Similarly, in a study by Jakubczyk et al. [58], most of the antioxidant substances in Matcha green tea were observed in infusions prepared at a higher temperature of 90 °C.

In this study, basil infusions after 2 h of storage under atmospheric conditions became darker, redder, and usually yellower, unlike infusions made at 85 °C. All the above-mentioned results suggest that the darker color of samples processed at lower temperatures results from the oxidation of polyphenolic compounds under the influence of polyphenyl oxidase (PPO) to o-quinones [59]. These react with other quinones, phenolic compounds, proteins, and amino acids to form brown complexes. PPO activity decreased with increasing temperature and showed very low activity at 75 °C [59,60].

The sous-vide method (*p* ≤ 0.05) resulted in darker, less green, and less yellow basil leaves than fresh and traditional steeping. The total color difference (ΔE) of sous-vide treated leaves was higher than for conventional treatments. The discussion on green vegetable color is a complex issue as many parameters and culinary procedures are applied by researchers. The less green color of sous-vide processed plants in comparison to traditionally treated plants was also reported by Martínez-Hernández et al. [61] in their study on broccoli, and in Rinaldi et al. [62] who studied Brussels sprouts. Moreover, in the case of sous-vide green vegetables, reduction in cooking temperature from 100 °C to 90 °C resulted in a decreased value of the parameter a* [63]. 

Cooking and storage also reduced the degree of green kale, which decreased with increasing cooking time [64]. This may be related to the degradation of chlorophyll during storage and the leaching of the color substances into the water during cooking. The study also found a lower color intensity of cooked kale than raw kale. Pero et al. [65] showed that the a */b * ratio, which is a validated indicator of the green color intensity in broccoli, increased in the initial phase of the process with the set temperature, probably because of the higher rate of water evaporation from the raw material. Further treatment leads to the loss of chlorophyll and its transformation into derivative compounds depending on temperature and pH. Armesto et al. [64] also found a high correlation between greenness (a* value) and total chlorophyll content. 

Vacuum conditions, no-water environment, and mild temperature of the sous-vide samples (60 °C) favored a lower transformation of chlorophyll (green color) to pheophytin (olive green color), through the magnesium substitution of the chlorophyll by hydrogen [66]. The highest amounts of pheophytin were found in samples cooked in boiling water (100 °C), cooked in water at a temperature of 85–90 °C, and in sous-vide samples [63]. 

Different conclusions were reached by Lafarga et al. [67,68], who reported no differences between the color of sous-vide and steamed broccoli, as well as Iborra-Bernad et al. [69,70], who did not observe any color difference between boiled, cook-vide, and sous-vide green beans pods.

## 4. Materials and Methods

### 4.1. Study Design

**Figure 2:**
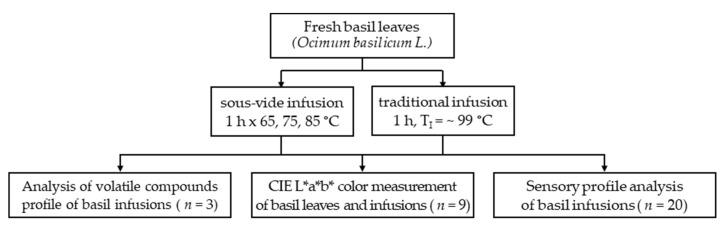
Study design (T_I_—initial temperature).

The study had a three-stage design (Figure 2). The first stage was an assessment of the effect of various temperatures for the sous-vide method on odor and flavor profile of basil aqueous infusions (*n* = 20). In the second stage, the color of basil leaves and infusion (*n* = 9) were evaluated. The third part of study was an analysis of the volatile compounds profile of basil infusions (*n* = 3), (Figure 2).

### 4.2. Material

The material was fresh basil (*Ocimum basilicum* L.), derived from commercial hydroponic cultivation (Baziółka, Swedeponic Polska Sp.z o.o., Kraśnicza Wola, Poland). The basil was kept in storage (10–15 °C) and used within 3 days. Plants cut off at 1.5 cm from the ground were rinsed, drained, and then the leaves were collected for further research.

Infusion preparation—As fresh herbs are usually a flavor source for plant- and animal-origin products, various cooking temperatures (65, 75, and 85 °C) and a constant time (1 h) for the sous-vide method were used to determine the effect of temperature on the profile of the main flavor notes. Temperatures of 65–75 °C correspond to the temperature range used in sous-vide cooking of meat and fish, while 85 °C is used for most sous-vide processed vegetables. As a reference point, a traditional infusion was also prepared.

Traditional infusion—Fresh basil (20 g) was placed in a glass beaker (400 mL capacity), then 180 mL of demineralized water (HLP 2O, Hydrolab Polska, Wiślina, Poland) heated to 95 °C was poured in, covered with aluminum foil, and left at room temperature for 60 min. After this time, the samples were cooled for 5 min in an ice bath to 21 ± 1 °C. Then, the infusion was drained and subjected to further stages of research.

Sous-vide infusions—Fresh herb (20 g) was placed in a thermostable polyethylene-polyamide pouch (130 × 230 mm), poured with a carrier (180 mL) at room temperature, vacuum packed in a vacuum packaging machine (No 691310, Stalgast, Warsaw, Poland), and placed in a sous-vide water bath (No 225448, Hendi, Gądki, Poland) preheated either to 65, 75, or 85 °C. After 60 min, the vacuum bags were placed in an ice bath, cooled to 21 ± 1 °C, strained, and subjected to the next stages of research.

### 4.3. Methods

#### 4.3.1. Analysis of Volatile Compounds

To determine the profile of volatile compounds released from basil water infusions, a headspace solid-phase microextraction (HS-SPME) method coupled to gas chromatography/mass spectrometry were used. Samples of fresh basil and traditional infusions were placed respectively in glass vials (20 mL) and glass containers (200 mL) with screw caps. Samples of sous-vide basil infusions were placed in dedicated bags for sous vide vacuum packaging. All samples before SPME extraction were conditioned for 20 min at 35 °C using a magnetic stirrer with heating function. A preconditioned (270 °C, 60 min), three-phase DVB/CAR/PDMS fiber (Supelco™ Analytical, Merck Life Science Sp. z.o.o., Poznań, Poland) was placed into headspace of given sample for 10 min at 35 °C, and after this period the fiber was transferred to gas chromatograph injection port set to 250 °C and working in split mode (1:20) for period of 2 min.

Chromatographic analysis and identification of volatile compounds was carried out using an Agilent 7890A (Agilent Technologies, Inc., Santa Clara, CA, USA) gas chromatograph coupled to a mass spectrometer (Inert XLMSD with Triple-Axis Detector, Agilent Technologies, Inc., Santa Clara, CA, USA) and equipped with an HP-5MS capillary column (30 m × 0.25 mm × 0.25 μm; Agilent Technologies Inc., Santa Clara, CA, USA). GC analysis parameters was as follows: the initial temperature of the column was set to 45 °C for period of 3 min, then it was raised to 200 °C at a rate of 5 °C/min, and maintained for 1 min. After this, temperature was increased to 240 °C at a rate of 15 °C/min and final temperature was maintained for 10 min. Helium was used as a carrier gas at a flow rate of 1 mL/min.

Mass spectra were obtained using electron ionization mode of 70 eV, the ion source temperature was set to 230 °C. The volatile compounds were identified by matching their mass spectra with database records of NIST and Wiley 8th spectra libraries (minimum 90% accuracy), and then confirmed by linear retention indices (LRI) calculated using a mixture of n-alkanes C7: C30 standards (Supelco™ Analytical, Merck Life Science Sp. z.o.o., Poznań, Poland). The quantities were expressed as percentages of the total identified signal.

#### 4.3.2. Sensory Analysis

##### Sensory Profiling

In order to determine the intensity of dominant odor and flavor attributes and to differentiate the color of samples, sensory profiling in accordance with the ISO 13299:2016 [71] procedure was performed. Fifteen sensory descriptors were selected and defined, and then assessed by panelists on a 10 cm unstructured graphic scale with word anchors at extreme values ranging from none to very intensive on the right. Selection of quality descriptors for quantitative descriptive analysis was carried out in accordance with the same procedure [71]. At first, panelists tasted samples and individually proposed the list of the sensory attributes for all sensory traits of each sample. Then during the panel members’ discussion led by a leader, the final list for assessment was selected. Finally, the sensory lexicon with word anchors was elaborated and agreed (Supplementary Data, Table S1). There were: fresh, basil, herbal, sweet, pungent, clove, and anise odor; color intensity; basil, fresh, and herbal flavor; sweet, and bitter taste; astringent sensation; and aftertaste. As the consistency of the infusions did not differ, its characterization was deliberately abandoned.

##### Sample Preparation and Presentation

Samples of basil infusions (15 mL) were served at room temperature (21 ± 1 °C) in transparent plastic containers (30 mL) with a lid, then coded with a 3-digit number and served to experts in random order. Natural water and wheat bread was provided as a taste neutralizer between samples.

##### Subjects and Testing Conditions

The evaluation was carried out on a group of 10 expert panelists, qualified [72] and experienced in sensory analysis, in two separate sessions with sufficient relaxation time in between. The trained panel consisted of 2 males and 8 females aged 30–55 years old.

The analysis was performed in an accredited sensory laboratory (contract No AB 564) fulfilling the requirements of the ISO standard [73] and equipped with individual test booths and sensory software ANALSENS.

#### 4.3.3. Instrumental Color Evaluation

Instrumental color measurements using spectrophotometer (CM-2300d, Konica-Minolta GmbH, Langenhagen, Germany) were carried out both basil leaves and its infusions. The equipment was set up with D_65_ standard illuminate (10° observer angle) and calibrated using a standard white plate (Minolta Technical Note 1994). In the case of leaves, the measurements were performed in nine replicates both before and after each heat treatment. Color of the infusions was measured using a 50 × 38 mm CM-APP plastic cuvette with an optical path length of 20 mm. Results were expressed as L* (lightness), a*(redness/greenness), and b* (yellowness/blueness) in the CIE Lab system.

Differences (Δ) between given coordinates of leaves were calculated by subtracting the colorimetric values of heat-treated sample from the raw material values. The value of color saturation delta chroma (ΔC) was calculated using (ΔC) = √((Δa*)^2^ + (Δb*)^2^) equation, the chroma with (Intensity of color): C* = √[(a*)^2^ + (b*)^2^] equation, while the value of total color difference (ΔE) with ΔE^*^_ab_= √[(ΔL*)^2^ + (Δa*)^2^ + (Δb*)^2^] equation.

#### 4.3.4. Statistical Analysis

One-way analysis of variance (ANOVA) with Fisher’s Least Significant Difference (LSD) post hoc test was applied to evaluate the significance of differences in volatile compounds composition, sensory profile, and color. Pearson correlation coefficients were calculated to relate the results of sensory profiling with the volatile compounds composition.

The statistical analysis was performed using the STATISTICA software version 13.3 PL package (StatSoft, Kraków, Poland) and considered as significant at the level of the materiality of 0.05. Interpretation of the sensory results obtained was carried out using the Principal Component Analysis (PCA) in accordance with [74].

## 5. Conclusions

The profile of volatile compounds of basil aqueous infusions differed significantly depending on the applied steeping methods. The hypothesis that the volatile compound profile of herbs processed with the sous-vide method is superior to conventionally infused profiles has not been confirmed. Our findings show that conventionally steeped infusions were more similar to the sous-vide infusion cooked at the lowest temperature SV_65_, while SV_75_ was more similar to SV_85_. Long cooking at higher temperatures results in a pronounced change in aroma composition. This study did not demonstrate a significant effect of the sous-vide cooking temperature on the odor and flavor profile evaluated by the panelists. The overall sensory impression of herbs is probably more favorable when assessed on common food matrices (e.g., meat, and vegetables). Due to varied odor activity values of volatiles and non-significant differences in sensory analysis, as well as limited data on the effect of the sous-vide method on the quality of herbs and complex process of creating flavor compounds, it cannot be concluded that the sous-vide method contributes better taste and aroma of herbs than conventional cooking methods Therefore, further research involving various factors (time–temperature combination, herb concentration, food matrices used) and other methods of analysis (e.g., Gas-Chromatography-Olfactometry, e-tongue, and e-nose) is needed.

## Figures and Tables

**Table 1:** Effect of various heat treatment methods on the volatile profile of basil infusions.

Volatile Compound	Odor Evocations acc. to [28]	LRI	LRI d.b.	Fresh	Sous-Vide Method			Traditional T_100_
					SV_65_	SV_75_	SV_85_	
				Relative Percentage Content (%) $\bar{x}$ *± SE*				
Dimethyl sulfide	cabbage, sulfur, gasoline	N/D	521	0.42 ± 0.15 ^a^	0.46 ± 0.13 ^a^	0.32 ± 0.01 ^a^	0.32 ± 0.01 ^a^	0.12 ± 0.01 ^a^
Hexane	-	801	800	0.12 ± 0.10 ^a^	-	-	-	0.0 ^a^ ± 0.10 ^a^
3-hexenal	leafy, green	803	803	0.16 ± 0.01	-	-	-	-
(E)-2-Hexenal	-	852	854	0.27 ± 0.12 ^a^	-	-	-	0.22 ± 0.10 ^a^
3-Hexen-1-ol	freshly cut grass	855	853	6.20 ± 0.48 ^b^	-	0.05 ± 0.01 ^a^	-	0.18 ± 0.04 ^a^
(E)-2-hexen-1-ol	green fruity	866	867	0.71 ± 0.17	-	-	-	-
1-Hexanol	flower, green	869	867	0.94 ± 0.23	-	-	-	-
Tricyclene	-	920	924	0.01 ± 0.00 ^a^	-	0.02 ± 0.00 ^b^	-	-
α-Thujene	wood, green, herb	927	927	0.25 ± 0.04 ^a^	0.32 ± 0.04 ^b^	0.66 ± 0.02 ^b^	0.48 ± 0.01 ^b^	0.29 ± 0.05 ^a^
α-Pinene	pine, turpentine	932	934	1.69 ± 0.13 ^a^	2.10 ± 0.49 ^a^	3.16 ± 0.09 ^b^	2.95 ± 0.01 ^b^	2.33 ± 0.15 ^a^
Camphene	camphor	947	948	0.55 ± 0.07 ^a^	0.62 ± 0.10 ^a^	1.04 ± 0.03 ^b^	1.07 ± 0.10 ^b^	0.84 ± 0.06 ^a,b^
Sabinene	pepper, turpentine, wood	972	972	2.38 ± 0.17 ^a^	2.65 ± 0.16 ^a^	3.11 ± 0.09 ^a^	2.97 ± 0.60 ^a^	3.97 ± 0.39 ^a^
β-pinene	pine, resin, turpentine	975	974	3.66 ± 0.23 ^a^	3.46 ± 0.54 ^a^	5.25 ± 0.15 ^b^	5.23 ± 0.18 ^b^	5.17 ± 0.37 ^b^
1-Octen-3-ol	mushroom	981	980	0.42 ± 0.08 ^a^	0.94 ± 0.14 ^b^	0.21 ± 0.01 ^a^	-	-
3-Octanone	nut	988	988	0.20 ± 0.06 ^b^	0.07 ± 0.01 ^a^	0.04 ± 0.00 ^a^	-	0.05 ± 0.03 ^a^
β-Mircene	balsamic, spicy	991	992	4.10 ± 0.17 ^a^	4.78 ± 0.27 ^a^	8.59 ± 0.25 ^b^	5.74 ± 0.32 ^a^	10.03 ± 0.9 ^b^
Decane	alkane	1000	1000	-	0.17 ± 0.03 ^a^	0.09 ± 0.02 ^a^	0.12 ± 0.03 ^a^	-
α-Phellandrene	turpentine, mint	1003	1003	0.35 ± 0.06 ^a^	-	-	-	1.16 ± 0.08 ^b^
Octanal	fat, soap, lemon	1004	1007	-	1.11 ± 0.10 ^a^	1.23 ± 0.04 ^a^	1.18 ± 0.032 ^a^	-
δ 3-carene	lemon, resin	1008	1008	1.44 ± 0.19 ^c^	0.47 ± 0.04 ^a^	0.66 ± 0.02 ^a^	0.85 ± 0.19 ^a,b^	1.02 ± 0.09 ^b^
α-Terpinene	lemon	1016	1015	0.22 ± 0.04 ^a^	0.31 ± 0.02 ^b,c^	0.42 ± 0.01 ^c^	0.53 ± 0.04 ^c^	0.32 ± 0.05 ^a,b^
2-Hexen-1-ol, acetate	-	1119	1119	0.04 ± 0.01	-	-	-	-
o-Cymene	lavender and cypress	1021	1021	0.01 ± 0.01	-	-	-	-
p-Cymene	gasoline, citrus	1024	1023	0.16 ± 0.03 ^a^	0.12 ± 0.00 ^a^	0.19 ± 0.01 ^a^	0.20 ± 0.04 ^a^	0.16 ± 0.03 ^a^
Limonene	sweet, citrus	1028	1028	3.05 ± 0.26 ^b^	2.01 ± 0.02 ^a^	2.74 ± 0.08 ^a^	1.97 ± 0.49 ^a^	5.09 ± 0.26 ^b^
Eucalyptol	eucalyptus, herb	1030	1030	24.91 ± 1.23 ^b^	39.44 ± 1.03 ^c^	26.53 ± 0.77 ^b^	30.30 ± 4.19 ^b^	14.21 ± 3.08 ^a^
cis-Ocimene	citrus, green, wood, terpene	1039	1040	0.31 ± 0.01 ^a^	0.24 ± 0.02 ^a^	0.47 ± 0.01 ^b^	0.41 ± 0.02 ^b^	0.60 ± 0.04 ^c^
trans-Ocimene	herb	1049	1050	11.17 ± 0.46 ^b^	7.65 ± 0.42 ^a^	10.61 ± 0.31 ^b^	9.03 ± 0.53 ^a^	16.66 ± 1.03 ^c^
γ-Terpinene	gasoline, turpentine	1058	1058	0.27 ± 0.04 ^a^	0.42 ± 0.02 ^b^	0.64 ± 0.02 ^c^	0.67 ± 0.06 ^c^	0.46 ± 0.06 ^b^
Sabinene hydrate trans	wood, balsamic	1067	1067	0.20 ± 0.01 ^b^	0.20 ± 0.02 ^b^	0.06 ± 0.01 ^a^	-	0.17 ± 0.03 ^b^
1-Octanol	forest, lemon, fatty	1080	1078	0.07 ± 0.01 ^b^	0.04 ± 0.00 ^a^	0.03 ± 0.00 ^a^	-	-
Terpinolene	pine, lemon, lime	1088	1088	3.57^b^ ± 0.58 ^b^	1.63 ± 0.16 ^a^	2.12 ± 0.06 ^a^	2.26 ± 0.31 ^a^	4.43 ± 0.33 ^b^
Undecene	alkane	1092	1092	-	0.02 ± 0.02 ^a^	-	-	0.13 ± 0.13 ^a^
β-Linalool	flower, lavender	1100	1100	4.96 ± 1.34 ^a^	13.89 ^c^ ± 1.8	8.01 ± 0.23 ^b^	9.51 ± 1.0 ^b^	9.40 ^b^ ± 0.41
Nonanal	fat, citrus, green	1107	1104	-	0.16 ± 0.02 ^a^	-	-	0.16 ± 0.01 ^a^
p-Mentha-1,3,8-triene	turpentine	1111	1113	0.02 ± 0.01 ^a^	-	0.02 ± 0.00 ^a^	-	0.02 ± 0.01 ^a^
Methyl caprylate	fruity, green	1125	1125	-	-	0.04 ± 0.00 ^a^	0.03 ± 0.03 ^a^	0.06 ± 0.00 ^a^
Allo-Ocimene		1128	1130	-	-	0.31 ± 0.01	-	-
Cosmene	bay leafy	1129	1134	0.12 ± 0.01 ^a^	0.10 ± 0.01 ^a^	-	0.14 ± 0.08 ^a^	0.15 ± 0.01 ^a^
Camphor	camphor	1143	1143	2.40 ± 0.27 ^b^	2.51 ± 0.33 ^b^	1.90 ± 0.05 ^b^	1.84 ± 0.016 ^b^	1.27 ± 0.16 ^a^
Borneol	camphor	1165	1165	0.20 ± 0.04 ^a^	0.34 ± 0.06 ^a^	0.17 ± 0.02 ^a^	0.21 ± 0.02 ^a^	0.20 ± 0.03 ^a^
Terpinen-4-ol	pine, pepper, wood	1178	1178	-	0.07 ± 0.01 ^a^	0.06 ± 0.02 ^a^	0.11 ± 0.01 ^b^	0.04 ± 0.01 ^a^
α-Terpineol	floral, lilac	1191	1187	-	0.33 ± 0.04 ^b^	0.15 ± 0.01 ^a^	0.15 ± 0.08 ^a^	0.25 ± 0.03 ^a,b^
Estragole	licorice, anise	1198	1199	0.22 ± 0.05 ^b^	-	0.13 ± 0.01 ^a^	-	0.27 ± 0.03 ^b^
Dodecane	alkane	1200	1200	-	0.95 ± 0.16 ^b^	0.45 ± 0.01 ^a^	0.62 ± 0.03 ^a^	-
Bornyl acetate	camphor, sweet, pine, herb	1287	1285	0.06 ± 0.01 ^a^	1.00 ± 0.08 ^c^	0.73 ± 0.02 ^b^	0.79 ± 02 ^b^	2.10 ± 0.09 ^d^
Tridecane	alkane	1300	1300	-	0.42 ± 0.08 ^b^	0.24 ± 0.01 ^a^	0.28 ± 0.00 ^a^	-
α-Cubebene	herb, wax	1351	1351	0.24 ± 0.12 ^a^	0.07 ± 0.01 ^a^	0.05 ± 0.00 ^a^	0.02 ± 0.01 ^a^	0.21 ± 0.04 ^a^
Eugenol	clove, honey	1359	1360	0.51 ± 0.28 ^a^	1.86 ± 0.32 ^b^	0.80 ± 0.02 ^a^	1.47 ± 0.20 ^b^	1.39 ± 0.10 ^b^
Copaene	wood, spice	1377	1377	0.69 ± 0.07 ^c^	0.12 ± 0.02 ^a^	0.06 ± 0.00 ^a^	0.01 ± 0.00 ^a^	0.29 ± 0.07 ^b^
Methyl cinnamate	strawberry	1383	1386	-	-	-	-	0.01 ± 0.00
β-Elemene	herb, wax, fresh	1393	1392	0.86 ± 0.12 ^c^	0.11 ± 0.06 ^a^	0.10 ± 0.00 ^a^	0.06 ± 0.06 ^a^	0.50 ± 0.90 ^b^
β-Caryophyllene	wood, spice	1421	1420	0.33 ± 0.02 ^a^	0.04 ± 0.02 ^a^	0.02 ± 0.00 ^a^	0.22 ± 0.22 ^a^	0.09 ± 0.05 ^a^
α-Bergamotene	wood, warm, tea	1437	1436	1.37 ± 0.19 ^b^	0.13 ± 0.04 ^a^	0.07 ± 0.00 ^a^	0.02 ± 0.02 ^a^	0.18 ± 0.10 ^a^
α-Guaiene	wood, balsamic	1441	1439	1.25 ± 0.20 ^b^	0.14 ± 0.07 ^a^	0.10 ± 0.01 ^a^	0.03 ± 0.03 ^a^	0.23 ± 0.14 ^a^
α-Humulene	wood	1456	1454	1.09 ± 0.09 ^b^	0.15 ± 0.04 ^a^	0.12 ± 0.00 ^a^	0.11 ± 0.02 ^a^	0.29 ± 0.15 ^a^
β-Farnesene	forest, sweet	1458	1457	7.00 ± 0.88 ^a^	0.74 ± 0.22 ^b^	0.47 ± 0.02 ^b^	0.26 ± 0.04 ^b^	1.14 ± 0.31 ^b^
epi-bicyclosesquiphellandrene	-	1465	1470	0.33 ± 0.04 ^a^	0.06 ± 0.02 ^b^	0.09 ± 0.01 ^b^	0.02 ± 0.02 ^b^	0.10 ± 0.06 ^b^
Epizonarene	-	1476	1478	0.05 ± 0.01 ^b^	0.01 ± 0.01 ^a^	-	-	-
γ-Muurolene	herb, wood, spice	1479	1477	0.17 ± 0.03 ^b^	-	-	0.01 ± 0.01 ^a^	0.03 ± 0.02 ^a^
Germacrene D	wood, spice	1483	1483	3.23 ± 0.48 ^c^	0.38 ± 0.10 ^a^	0.17 ± 0.2 ^a^	0.17 ± 0.09 ^a^	1.35 ± 0.22 ^b^
β-Selinene	herb	1488	1488	0.13 ± 0.01 ^a^	0.02 ± 0.01 ^a^	-	-	0.02 ± 0.02 ^a^
β-Guaiene	wood, spice	1491	1492	0.08 ± 0.03	-	-	-	-
Isoeugenol methyl ether	flower	1497	1499	-	-	-	0.06 ± 0.05 ^a^	0.45 ± 0.45 ^a^
Bicyclogermacrene	green, wood	1499	1500	1.46 ± 0.20 ^b^	0.27 ± 0.06 ^a^	0.31 ± 0.01 ^a^	-	0.46 ± 0.24 ^a^
δ- Guaiene	sweet, wood	1508	1506	1.76 ± 0.32 ^b^	0.25 ± 0.06 ^a^	0.10 ± 0.01 ^a^	0.07 ± 0.04 ^a^	0.32 ± 0.17 ^a^
γ- Cadinene	wood	1516	1516	0.92 ± 0.16 ^b^	0.12 ± 0.03 ^a^	0.08 ± 0.01 ^a^	0.06 ± 0.04 ^a^	0.56 ± 0.21 ^b^
δ-Cadinene	herb, wood, thyme	1525	1524	0.37 ± 0.05 ^b^	0.05 ± 0.01 ^a^	0.05 ± 0.00 ^a^	0.02 ± 0.02 ^a^	0.09 ± 0.05 ^a^
Cubenene	-	1534	1532	0.08 ± 0.01 ^a^	-	-	-	0.04 ± 0.01 ^a^
Alcohols				8.34 ± 1.13 ^a^	0.97 ± 0.25 ^b^	0.29 ± 0.01 ^c^	0.00 ± 0.00 ^d^	0.18 ± 0.07 ^e^
Aldehydes				0.43 ± 0.30 ^a^	1.27 ± 0.22 ^b^	1.23 ± 0.06 ^b^	1.18 ± 0.55 ^b^	0.39 ± 0.15 ^a^
Aliphatic hydrocarbons				0.12 ± 0.16 ^a^	1.55 ± 0.49 ^b^	0.78 ± 0.04 ^c^	1.02 ± 0.08 ^d^	0.22 ± 0.16 ^a^
Esters				0.04 ± 0.02 ^a^	0.00 ± 0.00 ^b^	0.04 ± 0.00 ^a^	0.03 ± 0.02 ^a^	0.08 ± 0.01 ^c^
Ethers				0.64 ± 0.19 ^a^	0.46 ± 0.23 ^a,b^	0.45 ± 0.02 ^b^	0.38 ± 0.11 ^b^	0.83 ± 0.71 ^a^
Ketones				0.20 ± 0.11 ^a^	0.07 ± 0.01 ^b^	0.04 ± 0.00 ^d^	0.00 ± 0.00 ^c^	0.05 ± 0.05 ^c,d^
Phenols				0.51 ± 0.49 ^a^	1.86 ± 0.56 ^b^	0.84 ± 0.04 ^a^	1.47 ± 0.35 ^b,c^	1.39 ± 0.17 ^c^
Terpenes				87.45 ± 1.10 ^a^	87.36 ± 5.11 ^a^	79.31 ± 3.97 ^b^	78.50 ± 7.82 ^b^	90.63 ± 10.70 ^c^

LRI—linear retention index calculated experimentally; LRI d.b.—linear retention index from the NIST database. N/D—no data; SE—standard error; ^a, b, c, d^—mean values marked by different letters in verses, differ significantly at *p* ≥ 0.05. SV_65_, SV_75_, SV_85_—temperature of sous-vide method.

**Table 2:** Effect of various heat treatment methods on the sensory profile of basil infusions.

Attribute	Sous-Vide Method			Traditional T_100_
	SV_65_	SV_75_	SV_85_	
	Intensity (0–10 c.u.) $\bar{x}$ ± SE			
Fresh odor	4.4 ± 0.5 ^a^	4.5 ± 0.5 ^a^	3.7 ± 0.5 ^a^	4.3 ± 0.5 ^a^
Natural basil odor	5.1 ^a^ ± 0.5 ^a^	5.2 ± 0.5 ^a^	4.8 ± 0.5 ^a^	4.8 ± 0.5 ^a^
Herbal odor	3.8 ^a^ ± 0.5 ^a^	3.7 ± 0.5 ^a^	3.2 ± 0.4 ^a^	3.3 ± 0.5 ^a^
Sweet odor	4.0 ^a^ ± 0.6 ^a^	3.2 ± 0.5 ^a^	3.0 ± 0.5 ^a^	3.5 ± 0.4 ^a^
Pungent odor	1.7 ^a^ ± 0.4 ^a^	1.9 ± 0.4 ^a^	2.0 ± 0.5 ^a^	1,6 ± 0.5 ^a^
Clove odor	2.3 ^a^ ± 0.4 ^a^	2.3 ± 0.3 ^a^	1.6 ± 0.2 ^a^	2.2 ± 0.3 ^a^
Anise odor	2.0 ^a^ ± 0.4 ^a^	1.8 ± 0.4 ^a^	1.4 ± 0.3 ^a^	1.6 ± 0.3 ^a^
Color intensity	7.0 ± 0.5 ^d^	6.0 ± 0.4 ^c^	1.2 ± 0.2 ^a^	4.1 ± 0.4 ^b^
Natural basil flavor	5.4 ± 0.4 ^a^	5.0 ± 0.5 ^a^	4.9 ± 0.6 ^a^	4.4 ± 0.6 ^a^
Fresh flavor	4.1 ± 0.8 ^a^	2.9 ± 0.8 ^a^	3.1 ± 0.7 ^a^	3.9 ± 0.6 ^a^
Herbal flavor	3.8 ± 0.6 ^a^	3.7 ± 0.4 ^a^	3.5 ± 0.5 ^a^	3.9 ± 0.5 ^a^
Sweet taste	2.2 ± 0.4 ^a^	1.6 ± 0.3 ^a^	1.8 ± 0.3 ^a^	2.1 ± 0.4 ^a^
Bitter taste	1.3 ± 0.3 ^a^	1. 7 ± 0.3 ^a^	2.0 ± 0.4 ^a^	2.0 ± 0.4 ^a^
Astringent sensation	2.0 ± 0.4 ^a^	2.3 ± 0.4 ^a^	2.4 ± 0.4 ^a^	2.5 ± 0.5 ^a^
Aftertaste	3.8 ± 0.6 ^a^	3.7 ± 0.5 ^a^	3.6 ± 0.6 ^a^	3.5 ± 0.5 ^a^

c.u.—conventional unit; SE—standard error; ^a, b, c, d^—different lowercase letters in rows indicate significant differences in color depending on the parameters applied at *p* ≤ 0.05.

**Table 3:** The color of basil leaves and infusions measured instrumentally.

Color	Fresh		Sous Vide Method $\bar{x}$ *± SE*			Traditional T_100_
			SV_65_	SV_75_	SV_85_	
Color of infusion after preparation	L	-	26.79 ± 0.83 ^a,A^	30.25 ± 1.20 ^b,A^	36.77 ± 0.94 ^c,A^	32.82 ± 0.77 ^b,A^
	a	-	8.24 ± 0.74 ^c,A^	4.48 ± 0.75 ^b,A^	0.01 ± 0.31 ^a,A^	5.46 ± 0.44 ^b,A^
	b	-	26.97 ± 0.88 ^c,A^	21.91± 1.05 ^b,A^	15.49 ± 0.52 ^a,A^	17.43 ± 0.48 ^a,A^
Color of infusion after 2h	L	-	16.51 ± 0.46 ^a,B^	16.97 ±0.26 ^a,B^	36.24 ± 0.46 ^c,A^	25.60± 0.62 ^b,B^
	a	-	17.11 ± 0.31 ^c,B^	16.59 ± 0.18 ^c,B^	0.22 ± 0.31 ^a,A^	10.08 ± 0.17 ^b,B^
	b	-	23.24 ± 0.46 ^b,B^	24.02 ± 0.53 ^b,A^	15.30 ± 0.46 ^a,A^	24.08 ± 0.23 ^b,B^
Color of leaves	L	34.89 ^c^ ± 0.83	9.8 ± 1.00 ^a^	9.6 ± 1.41 ^a^	8.4 ^a^ ± 0.69 ^a^	18.8 ± 0.88 ^b^
	a	−13.95 ^d^ ± 1.57	−5.6 ± 1.01 ^a^	−3.8 ± 0.51 ^b^	−0.3 ± 0.32 ^c^	−11.7 ± 0.75 ^d^
	b	45.14 ^c^ ± 1.62	16.6 ± 1.70 ^a^	15.4 ± 2.77 ^a^	14.2 ^a^ ± 1.18 ^a^	31.6 ± 1.49 ^b^
	C	46.99	17.48	15.90	13.87	34.03
	ΔC	-	29.54	31.11	33.80	13.20
	ΔE	-	38.56	39.92	42.88	20.42

SE—standard error; ^a, b, c, d^—different lowercase letters in rows indicate significant differences in color depending on the parameters applied at *p* ≤ 0.05; ^A, B^—different uppercase letters in the column denote significant differences in infusion color after 2 h storage.

**Table 4:** List of volatile compounds identified in basil infusions by other authors.

Volatile Compound *	Authors	Volatile Compound *	Authors
Dimethyl sulfide	-	p-Mentha-1,3,8-triene	-
Hexan	-	Octanoic acid, methyl ester	-
3-hexenal	-	Allo-Ocimene	-
(E)-2-Hexenal	2	Cosmen	-
3-Hexen-1-ol	2	Camphor	1,2,3,4,5
(E)-2-hexen-1-ol		Borneol	1,2,4,5
1-Hexanol	-	Terpene-4-ol	1,5
α-Thujene	1,5	Dodekan	-
Tricyclene	-	α-Terpineol	1,2,3,4,5
α-Pinene	1,2,4,5	Estragole	1,2,3,4
Camphene	1,2	Bornyl acetate	1,2,3,4,5
Sabinene	1,2,3	Tridecane	-
β-pinene	1,2,4,5	α-Cubebene	-
1-Octen-3-ol	3,4	Eugenol	1,2,4
β-Mircene	1,2,5	Copaene	1,2,
3-Octanone	-	Methyl cinnamate	1,2,3,5
α-Phellandrene	2	β-Elemene	1,2,3,5
Octanal	-	β-Caryophyllene	1,2,3,4,5
Decane	-	α-Bergamotene	1,2,3,4,5
α-Terpinene	1	α-Guaiene	1,2,5
δ 3-carene	-	α-Humulene	1,2,3,4
o-Cymene	2	β-Farnesene	1,2,3,4
p-Cymene	-	epi-bicyclosesquiphellandrene	-
Limonene	1,4,5	Epizonarene	-
Eucalyptol	1,3,4,5	γ-Muurolene	-
cis-Ocimene	2,3,4	Germacrene D	1,2,3,4,5
trans-Ocimene	1,	β-Selinene	-
γ-Terpinene	1,3	β-Guaiene	1
Sabinene hydrate trans	1,4	Isoeugenol methyl ether	-
1-Octanol	2	δ-Cadinene	-
Terpinolene	2,4	Bicyclogermacrene	2
Undecene	-	Cubenene	-
β-Linalool	1,2,3,5	δ-Guaiene	-
Nonanal	-	δ-Cadinene	1,4,5

* volatile compounds identified in our study; 1—Beatovic et al. [37]; 2—Tarchoune et al. [36]; 3—Avetisyan et al. [38]; 4—Jordán et al. [39]; 5—Adams et al. [33].

## Data Availability

Not applicable.

## Supplementary Materials

The following are available online. Table S1: Sensory lexicon used in the study.

## Sample Availability

Samples are not available from the authors.
