# Supplementary material for: Fresh Basil Infusion: Effect of Sous-Vide Heat Treatment on Their Volatile Composition Profile, Sensory Profile, and Color
Source: Molecules. 2021 Dec 21;27(1):5. doi: 10.3390/molecules27010005 (PMC8746197; doi:10.3390/molecules27010005)
Supplement: Supplementary file 1 [file molecules-27-00005-s001.zip › molecules-1509514-supplementary.pdf]

Table S1. Sensory lexicon used in the study

descriptor	definition	word anchors
	**odor**	
fresh	fresh, characteristic of cut herbs e.g. mint, cress	none - very strong
natural basil	aroma characteristic for fresh sweet basil, intense	none - very strong
herbal	characteristic of dried basil or herbal mixture of provencal herbs	none - very strong
sweet	pleasant, sweet-smelling aroma, typical of caramel or hard candy, with hints of licorice	none - very strong
pungent	strong, biting (acrid) aroma	none - very strong
clove	characteristic of clove buds: fruity, medicinal, minty, woody notes (reference material)	none - very strong
anise	characteristic of anise (reference material)	none - very strong
	**color**	
color intensity	intensity of the infusion color ranging from pale straw color to dark beige	none - very strong
	**flavor / taste/ trigeminal sensation**	
natural basil flavor	typical of fresh basil, sweet, refreshing, slightly peppery and licorice-anise	none - very strong
fresh flavor	characteristic of mint, freshly cut grass, unripe fruit.	none - very strong
herbal flavor	characteristic of dried basil or herbal mixture of Provencal herbs	none - very strong
sweet taste	basic taste quality, it does not need to be defined	none - very strong
bitter taste	basic taste quality, it does not need to be defined	none - very strong
astringent sensation	perception of a puckering sensation in the mouth when the sample is spread over the palate. caused by substances such as tannins and alum and long lasting in the mouth.	none - very strong
aftertaste	general sensation appearing after the disappearance of the taste stimulus.	none - very strong
